# Inter-observer variation in gross tumour volume delineation of oesophageal cancer on MR, CT and PET/CT

**DOI:** 10.2478/raon-2024-0043

**Published:** 2024-10-04

**Authors:** Ajra Secerov-Ermenc, Primoz Peterlin, Franc Anderluh, Jasna But-Hadzic, Ana Jeromen-Peressutti, Vaneja Velenik, Barbara Segedin

**Affiliations:** Department of Radiation Oncology, Institute of Oncology Ljubljana, Ljubljana, Slovenia; Faculty of Medicine, University of Ljubljana, Ljubljana, Slovenia

**Keywords:** oesophageal cancer, gross tumour volume, positron emission tomography, magnetic resonance

## Abstract

**Background:**

The aim of our study was to assess the inter-observer variability in delineation of the gross tumour volume (GTV) of oesophageal cancer on magnetic resonance (MR) in comparison to computed tomography (CT) and positron emission tomography and CT (PET/CT).

**Patients and methods:**

Twenty-three consecutive patients with oesophageal cancer treated with chemo-radiotherapy were enrolled. All patients had PET/CT and MR imaging in treatment position. Five observers independently delineated the GTV on CT alone, MR alone, CT with co-registered MR, PET/CT alone and MR with co-registered PET/CT. Volumes of GTV were measured per patient and imaging modality. Inter-observer agreement, expressed in generalized conformity index (CIgen), volumetric conformity index (VCI), planar conformity index (PCI) and inter-delineation distance (IDD) were calculated per patient and imaging modality. Linear mixed models were used for statistical analysis.

**Results:**

GTV volume was significantly lower on MR (33.03 cm^3^) compared to CT (37.1 cm^3^; p = 0.002) and on PET/CT MR (35.2 cm^3^; p = 0.018) compared to PET/CT (39.1 cm^3^). The CIgen was lowest on CT (0.56) and highest on PET/CT MR (0.67). The difference in CIgen between MR (0.61) and CT was borderline significant (p = 0.048). The VCI was significantly higher on MR (0.71; p = 0.007) and on CT MR (0.71; p = 0.004) compared to CT (0.67). The PCI was significantly higher on CT MR (0.67; p = 0.031) compared to CT (0.64). The largest differences were observed in the cranio-caudal direction.

**Conclusions:**

The highest inter-observer agreement was found for PET/CT MR and the lowest for CT. MR could reduce the difference in delineation between observers and provide additional information about the local extent of the tumour.

## Introduction

Oesophageal cancer is the 11th most common cancer and the 7th leading cause of cancer death worldwide.^1^ It is characterised by high mortality, poor prognosis and a variable geographical distribution.^2^ Surgery and radiotherapy play an important role in both limited and locally advanced disease.^3,4,5,6,7^ Accurate tumour delineation is crucial in radiotherapy planning to ensure adequate target coverage and local control of the disease, which can also impact on disease free survival and overall survival.^8^ Computed tomography (CT) is the standard imaging modality for radiotherapy treatment planning in oesophageal cancer. However, it can overestimate the length of the tumour.^9^ Other imaging modalities could therefore have a role in radiotherapy treatment planning, in particular, positron emission tomography (PET) and magnetic resonance imaging (MR).^8,10,11^

18-F-fluorodeoxyglucose (FDG)-PET/CT is considered an essential diagnostic method for the initial staging of oesophageal cancer, because of its ability to detect metastatic disease, including lymph node metastases (LNM), with 66% sensitivity and 96% specificity.^12,13^ FDG-PET/CT seems superior to CT, especially in the detection of LNM, consequently, it is essential when determining nodal clinical target volume (CTV) in the elective or involved-field irradiation.^14,15,16^ On the other hand, PET-based segmentation algorithms or PET manual contouring of the primary tumour did not show a good correlation compared to CT imaging after clipping the cranial and caudal border of the tumour.^17^ However, studies comparing the length of the tumour on preoperative FDG-PET/CT scans with histopathological specimens after surgery showed a good correlation.^18,19,20^ Some studies showed improved inter-observer variability in the delineation of gross tumour volume (GTV) on PET/CT, while others did not confirm it.^21,22,23,24,25,26^ GTV delineation on FDG-PET/CT imaging thus remains controversial.

MR is not yet an established method for radiotherapy treatment planning for oesophageal cancer, but it is promising because of its excellent soft tissue contrast. Over the past decade, several technical innovations have reduced image artefacts, such as the use of automatic gating navigators or multi-channel receiver coils.^27^ The longitudinal length of GTV measured on diffusion-weighted MR (DWI) correlated more precisely with the length of the histopathological specimen compared to CT or T2-weighted MRI (T2 MRI).^28^ MR-based GTV delineation of oesophageal cancer was feasible, with inter-observer variability comparable to FDG-PET/CT.^29^ However, further studies are needed.

The aim of our study was to assess the interobserver variability in delineation of the GTV in oesophageal cancer on MRI in comparison to CT and PET/CT.

## Patients and methods

The study was approved by the National Medical Ethics Committee of Slovenia (No. 0120-620/2019/3) on 21 January 2020 and in accordance with the Declaration of Helsinki. The study was registered in the ClinicalTrials.gov database (NCT05611658). Written informed consent was obtained from all patients.

### Patients

We prospectively enrolled 23 patients with locally advanced oesophageal cancer from April 2020 to May 2021. Patients had to meet the following inclusion criteria: locally advanced oesophageal cancer, Siewert I or II for distal oesophageal tumours, planned preoperative or definitive chemoradiotherapy, no contraindications for MR. We included 20 men and 3 women with an average age of 61 years.

Patient characteristics are listed in Table 1.

**TABLE 1.** Characteristics of the patients with oesophageal cancer enrolled in the study

**Case**	**Location – third**	**Histology**	**Treatment**	**Stage**	**Gender**	**Age**
**1**	Proximal	SCC	definitive	T3N0M0	M	62
**2**	Proximal	SCC	definitive	T3N0M0	M	66
**3**	Distal	AC	preoperative	T3N1M0	M	64
**4**	Middle	SCC	definitive	T3N1M0	M	68
**5**	Proximal	SCC	definitive	T3N2M0	F	60
**6**	Proximal	SCC	definitive	T3N1M0	M	57
**7**	Distal	AC	preoperative	T3N1M0	M	66
**8**	Proximal	SCC	definitive	T3N1M0	M	64
**9**	Distal	AC	definitive	T2N0M0	M	81
**10**	Distal	AC	preoperative	T3N2M0	M	35
**11**	Proximal	SCC	definitive	T3N0M0	M	58
**12**	Distal	SCC	preoperative	T3N1M0	M	61
**13**	Proximal	SCC	definitive	T3N0M0	M	63
**14**	Middle	SCC	preoperative	T3N0M0	M	54
**15**	Distal	AC	preoperative	T3N0M0	M	64
**16**	Middle	SCC	definitive	T3N1M0	F	83
**17**	Distal	AC	preoperative	T3N1M0	M	58
**18**	Proximal	SCC	definitive	T3N0M0	M	42
**19**	Distal	AC	preoperative	T3N0M0	M	75
**20**	Proximal	SCC	definitive	T3N0M0	M	70
**21**	Proximal	SCC	definitive	T3N2M0	F	46
**22**	Middle	SCC	preoperative	T3N0M0	M	53
**23**	Middle	SCC	preoperative	T2N2M0	M	67

AC = adenocarcinoma; F = female; M = male; SCC = squamous cell carcinoma

### Image acquisition

#### FDG-PET CT

All patients underwent a planning FDG-PET/CT scan in treatment position on Siemens BiographTM mCT 40 PET-CT simulator after standard preparation protocol. The activity of the intravenously administered 18F-FDG was 3.7 MBq/kg. After about 60 minutes, the CT scan was performed with the following settings: 120 kV, 200 mAs, 1 second rotation time, pitch of 0.8 and 3 mm slice thickness. An iodine intravenous contrast agent was administered before the CT scan. After CT, a PET scan was acquired 3-dimesionally with duration of 2 minutes per bed position.

#### MR

MR imaging was performed on a 1.5T Optima™ MR450w GE MR simulator (General Electric). Patients were scanned prior to radiotherapy in treatment position without intravenous contrast. We acquired T2-weighted images in the transverse plane with a slice thickness of 3 mm and diffusion-weighted images (DWI) for each patient.

### Target volume delineation and observers

Five radiation oncologists with at least 5 years of experience in the treatment of oesophageal cancer delineated the gross tumour volume (GTV). Contouring was performed using the Eclipse™ planning system (Palo Alto, California, USA). Initially, a meeting was organised with the aim of familiarising the observers with MR images of oesophageal cancer. Under the guidance of an experienced radiologist, they delineated the GTV on MR images of two pilot cases. The observers received relevant information about the location and characteristics of the tumour. GTV was contoured separately on different imaging modalities, as follows: CT, PET/CT, MR, CT with MR fusion and PET/CT with MR fusion. All the images in the study were anonymised.

GTV was defined as the visible tumour on imaging as the whole circumference of the oesophagus. Regional pathological lymph nodes were not included in the GTV. Contouring on different imaging modalities was performed after an interval of at least two weeks to minimise recollection of the previous images.

When contouring on the PET CT images, the observers delineated the GTV on the CT and corrected it according to the PET images if necessary. The GTVPET corresponded to 20% of the maximum standardized uptake value (SUV). The PET and CT images were then fused. The visible tumour was contoured as GTV and, if necessary, corrected taking into account the GTVPET. When contouring on the MR images, the observers delineated the GTV on the T2 MR and modified it using the DWI if necessary.

Observers were instructed to record the delineation time, image quality (good, moderate, poor) and difficulty of contouring the GTV in all imaging modalities (five-point scale: very difficult – very easy).

### Data analysis

GTV volumes were measured per observer, per patient and per imaging modality and average volumes were calculated per patient and per imaging modality. In order to assess contouring uncertainties, we calculated the generalized conformity index (CIgen), which is independent of the number of volumes analysed.^30^ The CIgen was calculated per patient and averaged over all patients per imaging modality.

$$\mathrm{CI}\text{gen}=\frac{\Sigma \text{pairs}\mathrm{ij}[\mathrm{Ai}\cap \mathrm{Aj}]}{\Sigma \text{pairs}\mathrm{ij}[\mathrm{Ai}\cup \mathrm{Aj}]}$$

In order to quantify the accuracy of the contouring, the deviations of the observers from the reference contours were analysed. The Contour analysis tool 2 (CAT 2) software and the associated methodology were used for the volumetric and distance-based calculations. The reference contour was calculated using the Simultaneous Truth and Performance Level Estimation (STAPLE) algorithm from the collection of the contours from all observers per patient and per imaging modality.^31^ Using the STAPLE delineations as a reference, the volumetric conformity index (VCI) - the ratio of the intersection and union of the test and reference volumes - of the pairs between the reference and each test delineation were averaged for each imaging modality. In addition, the planar conformity index (PCI) was calculated as the ratio between the intersection and union surface of the test and reference contours on each image slice and presented as a function of slice number for each patient.^32,33^ To assess cranio-caudal variation, we evaluated the mean distance of the caudal and cranial borders of the tumour for all observers between CT and MR, CT and MR CT, and PET/CT and PET CT MR. We recorded the number of slices and multiplied it by the slice thickness (3 mm).

**FIGURE 1.**
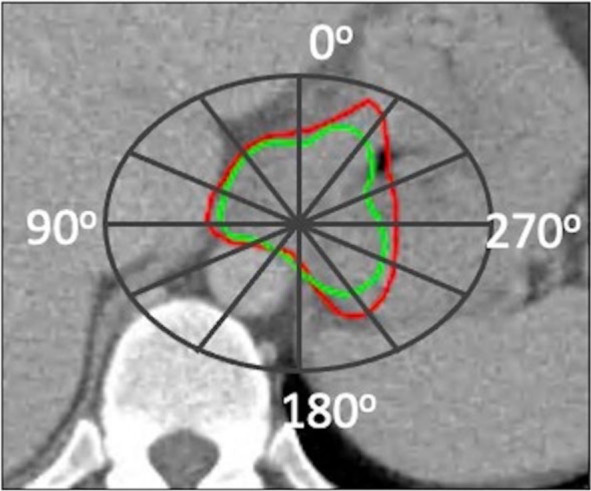
The coordinate system for spatial assessment of inter-delineation distances is projected on a single slice of imaging modality containing an example of GTV and divided in 12 angular segments (6 on the left and 6 on the right). Green line represents the reference contour, red line is the test contour.

For the analysis in the transverse plane, we calculated the inter-delineation distance (IDD), which is the shortest distance between the reference and test contours from the centre of mass in 72.5° angular segments, expressed in millimetres. Using the centre of mass as the origin, a coordinate system was defined and divided into 12 angular segments (6 on the right and 6 on the left) that were separated on each transverse slice of all imaging modalities: on the right (0–25°, 30–55°, 60–85°, 90–115°, 120–145° and 150–175°) and on the left (180–205°, 210–235°, 240–265°, 270–295°, 300–325° and 330–355°) (Figure 1).

We calculated the average IDD for all slices of the imaging methods, all observers and all test cases for each target volume. This method has been previously used and described in detail.^32,33,34^

#### Statistic analysis

Before starting the analysis, we calculated the sample size. Taking into account the data from previous studies, we calculated that we would need about 21 patients at a significance level of 0.05 and a statistical power of 0.8.^21,29,35^

The numerical variables were presented as means and standard deviations (SD). The correlation between CIgen and imaging modalities was tested with linear mixed models, patients were included as a random factor. When analyzing the association between VCI, PCI, GTV volume and imaging modalities observers were included as a random factor in addition to patients. The analysis was performed with R 4.3.2 using the libraries *lme4*, *lmerTest*, *emmeans* and *forestplot*.

## Results

The results are presented in Table 2.

**TABLE 2.** Mean volume of gross tumor volume (GTV), CIgen, VCI, PCI, IDD and standard deviation

	**CT (SD)**	**MR (SD)**	**CT MR (SD)**	**PET/CT (SD)**	**PET/CT MR (SD)**
**Volume (cm^3^)**	37.14 (35.66)	33.03 (30.40)	35.04 (32.58)	44.12 (39.10)	40.94 (35.16)
CIgen	0.56 (0.18)	0.61 (0.14)	0.61 (0.14)	064 (0.13)	0.67 (0.12)
VCI	0.67 (0.18)	0.71 (0.15)	0.71 (0.14)	0.74 (0.14)	0.77 (0.12)
PCI	0.64 (0.17)	0.67 (0.16)	0.67 (0.15)	0.71 (0.14)	0.73 (0.13)
IDD (mm)	1.39 (1.47)	1.44 (1.44)	1.70 (1.50)	1.68 (1.50)	1.60 (1.66)

CIgen = generalized conformity index; CT = computed tomography; ; CT MR = fusion of CT and MR; MR = magnetic resonance imaging; IDD = inter-delineation distance; PET/CT = positron emission tomography and CT; PET/CT MR = fusion of PET/CT and MR; VCI = volumetric conformity index; PCI = planar conformity index; SD = standard deviation

The mean GTV volume is lowest in MR (33.03 cm^3^) and highest in PET/CT (44.12 cm^3^). The difference in mean GTV volume between MR and CT (p = 0.002) and between PET/CT MR and PET/CT is statistically significant (p = 0.018).

The mean CIgen is lowest on CT (0.56) and highest for PET/CT MRI (0.67). The difference in mean CIgen between MR and CT is borderline significant (p = 0.048).

**FIGURE 2.**
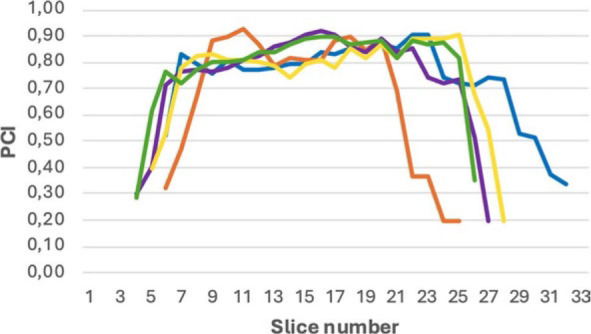
Mean planar conformity index (PCI) for the GTV as a function of slice number for all imaging modalities for case 22. Blue = computed tomography (CT); Green = fusion of PET/CT and MR; Red = magnetic resonance imaging (MR); Violet = positron emission tomography and CT (PET/CT); Yellow = fusion of CT and MR (CT MR); 1= most caudal slice; 33= most cranial slice.

**FIGURE 3.**
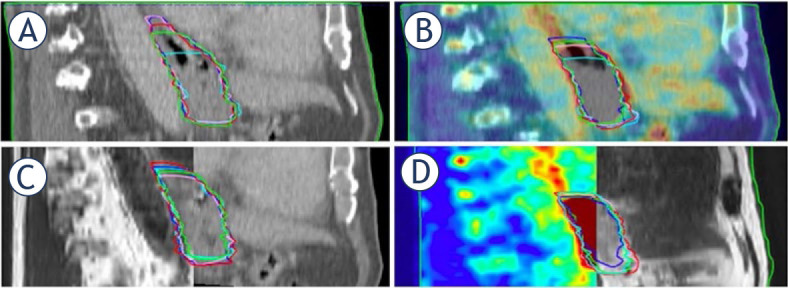
Delineation of the gross tumour volume (GTV) of all five observers of case 22, sagittal view. The variation in cranial border is highest on computed tomography (CT) and lowest on positron emission tomography (PET/CT) magnetic resonance (MR). (A) CT; **(B)** PET/CT; **(C)** fusion of CT and MR; **(D)** fusion of PET/CT and MR.

The VCI is on average the lowest on CT (0.67) and the highest on PET/CT MR (0.77). The difference in mean VCI between MR and CT (p = 0.007) and between CT and MR is statistically significant (p = 0.008). The PCI is on average the lowest in CT (0.64) and the highest in PET/CT MR (0.70). The difference in mean PCI between CT MR and CT is statistically significant (p = 0.031). We analysed the PCI per imaging modality as a function of the number of slices for each patient. In all imaging modalities, the variations were greatest caudally and cranially, while agreement was high in the middle of the target volumes. An example is shown in Figure 2 and 3.

The mean distance between the caudal border of the GTV on CT and MR, CT and CT MR, and PET/CT and PET/CT MR was 12.00 mm, 10.96 mm, and 4.57 mm, respectively. The mean distance between the cranial border of the GTV on CT and MR, CT and CT MR and PET/CT and PET/CT MR was 12.26 mm, 6.22 mm, 3.56 mm, respectively.

**FIGURE 4.**
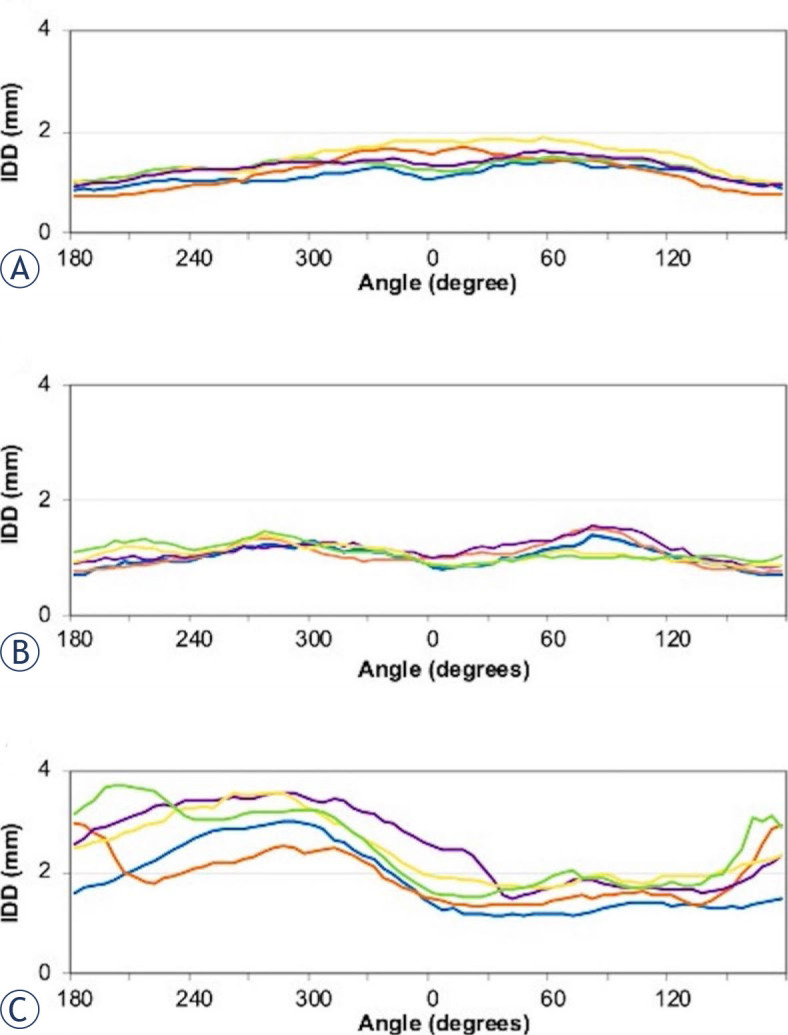
Mean inter-delineation distance (IDD) curves of the gross tumour volume (GTV) delineated on different imaging modalities as a function of the angle. The IDD is largest in tumours of the lower third and similar in upper and thirds. **(A)** Upper third of the oesophagus; **(B)** Middle third of the oesophagus; **(C)** Lower third of the oesophagus. Blue = computed tomography; Green = fusion of PET CT and MR. Red = magnetic resonance imaging; Violet = positron emission tomography and CT; Yellow = fusion of CT and MR

In the analysis in the transverse plane, we found that the IDD is smallest on CT and largest on CT-MR. Comparing the average values between the imaging modalities for each angular segment, CT has the lowest values at 0–25°, 30–55°, 60–85°, 90–115°, 150–175°, 180–205° and 330–355°, which is predominantly on the right lateral side, while MR has the lowest values in all other angular segments, predominantly on the left side. We recorded larger mean IDDs when delineation was performed using fused imaging modalities. The cases were divided into groups of patients according to the tumour location in the oesophagus (upper, middle and lower third). We found that IDDs were small in tumours in the upper and middle third of the oesophagus, in the range of 2 mm. In tumours in the lower third of the oesophagus, the IDDs were larger, especially in the angular segments on the left lateral side, up to 4 mm (Figure 4).

We also compared the difference in IDD between CT and MR, CT and MR CT, and PET CT and PET CT MR. Statistically significantly higher IDDs were observed mainly when comparing MR CT with CT sequences, at angular segments 180–205° (p = 0.030), 120–145° (p = 0.005), 60–85° (p = 0.009) and 30–55° (p = 0.003).

Image quality was rated good in 66%, 59%, 51%, 43% in 41% in PET/CT MR, PET/CT, CT MR, MR and CT, respectively. The difficulty of contouring was rated as easiest for PET CT MR (22% very easy and 5% very difficult) and most difficult for CT, with the highest rating of being very difficult (23%). In terms of contouring time, MR tends to have the lowest values compared to the other modalities with a median (Me) of 4.6 minutes (interquartile range (IQR) 4.1–5), followed by PET CT (Me 6.4; IQR 4.7–6.8), CT MR (Me 6.6; IQR 5.2–7.6), PET CT MR (Me 7; IQR 5.3–7.9) and CT (Me 7.2; IQR 6–8.4).

## Discussion

In our study, five observers contoured the GTV of oesophageal tumours of 23 patients treated with preoperative or definitive chemoradiotherapy on CT, PET/CT, MR, CT MR fusion and PET/CT MRI fusion.

The results showed that the mean GTV volume was smallest on MR and largest on PET/CT. MR significantly reduced the volume compared to CT and when fused with PET/CT compared to PET/CT alone. Similarly, Vollenbrock *et al.* compared GTV contouring of oesophageal cancer on PET/CT, MR and MR with DWI sequences and reported statistically significantly smaller volumes on MR with DWI compared to PET/CT and MR.^29^ Furthermore, the tumour length in the histopathological specimen correlated better with the length of the GTV contoured on DWI than with target volumes on CT or T2-MR, so we can assume that MR images with DWI sequences are closest to the ”ground truth” of tumour length.^28^ One of the possible reasons for larger GTV volumes on PET/CT is that FDG is not tumour specific and high FDG uptake could also be due to inflammation. Patients with oesophageal cancer often have erosive oesophagitis caused by alcohol consumption or gastro-oesophageal reflux. The inflammation of the oesophagus detected by FDG PET/CT correlates with the endoscopic findings.^36^ The inclusion of the FDG-avid area in the GTV may not represent only the tumour. Observers tend to delineate larger volumes on CT as well, mainly because of poor soft tissue contrast, especially in the cranio-caudal direction, as confirmed by histopathological correlation.^9^ Poor differentiation of tumour borders could lead to large cranio-caudal variation between observers. Fusion of MR with CT or PET/CT reduced the variation. However, even when the imaging modalities were fused, the cranio-caudal deviation remained large.

On the other hand, the uncertainty in contouring in the transverse plane in oesophageal cancer was low and probably not clinically significant. We analysed the IDD as a function of the angles in the transverse plane in relation to the thirds of the oesophagus due to the different anatomical conditions. In the upper and middle third, we observed small differences of less than 2 mm in all angular segments, while in the lower third the differences were up to 4 mm mainly in the left lateral angular segments. The oesophagus merges into the stomach in the distal part to the left side and it is more difficult to define the tumour borders in this area. Different fused imaging modalities did not reduce this uncertainty. Two other studies confirmed small variations in the transverse plane.^21,29^

Our study has some limitations. Firstly, the observers had no experience in contouring on MR for oesophageal cancer before the start of the study, which could explain the lower CIgen on MR compared to PET/CT. We tried to overcome this problem by organising a meeting with an experienced radiologist and contouring on two pilot cases. However, this probably remained a disadvantage as the observers had several years of experience with delineation on PET/CT. Vollenbrock *et al*. concluded that contouring on MR images is feasible despite lack of experience as they found similar inter-observer variability between MR and PET/CT.^29^ Secondly, the observers delineated the same cases multiple times, starting with CT and ending with PET/CT MR, so they could recall the anatomical features of the cases to some extent, which could represent bias. All the metrics of overlap, image quality and contouring difficulty were lowest/worst for CT and highest/best for PET/CT MR. To minimise recall of the images, at least 14 days (usually more) had elapsed between delineations, but this was probably not enough to overcome it completely. Thirdly, all observers come from the same institution. All had at least five years of experience, but some have learnt from others over the years, which could be the reason for the similarity of the contours and does not represent all real-life scenarios.

## Conclusions

In conclusion, the lowest inter-observer agreement was found for CT and the highest for PET/CT MR. The improvement in inter-observer agreement with fused imaging modalities is mainly due to the reduction of differences in the cranio-caudal direction, which may affect dose distribution and thus local control and side effects of radiotherapy treatment. MR and CT MR imaging reduced inter-observer variability compared to CT imaging alone, but not compared to PET/CT. Therefore, the use of MR for delineation could be recommended, especially for non-FDG-avid tumours or for patients with marked inflammation of the oesophagus, which can be assessed in scans prior to treatment. MR imaging provides additional information about the local extent of the tumour. As the number of patients with oesophageal cancer is not high, additional MR simulation would not represent a major financial burden, but optimisation of imaging protocols and further studies are required. Its use can minimise cranio-caudal variation, but more experience and appropriate training programmes are needed.
